# Chitotriosidase deficiency is not associated with human hookworm infection in a Papua New Guinean population

**DOI:** 10.1016/j.meegid.2007.07.010

**Published:** 2007-12

**Authors:** Andrew J. Hall, Rupert J. Quinnell, Andrew Raiko, Moses Lagog, Peter Siba, Shaun Morroll, Franco H. Falcone

**Affiliations:** a Immune Modulation Research Group, School of Pharmacy, University of Nottingham, Nottingham NG7 2RD, United Kingdom; b Institute of Integrative and Comparative Biology, University of Leeds, Leeds LS2 9JT, United Kingdom; c Papua New Guinea Institute of Medical Research, PO Box 60, Goroka EHP 441, Papua New Guinea

**Keywords:** Chitotriosidase, *CHIT1* mutation, *Necator americanus*, Malaria, Papua New Guinea

## Abstract

Human chitotriosidase (*CHIT1*) is a chitinolytic enzyme with suggested anti-fungal properties. Previous studies have suggested that chitotriosidase may also protect individuals against filarial nematode infections and malaria. A mutant allele, which renders chitotriosidase unstable and enzymatically inactive, is found at a frequency of >20% in Caucasians and other populations. This allele is found at much lower frequency in parts of West Africa where malarial and intestinal helminth infections are endemic. Here, we investigate whether there is a significant association between chitotriosidase genotype and the intensity of hookworm infection in 693 individuals from five villages in Papua New Guinea. Individuals were genotyped for chitotriosidase using a PCR-based assay. There was no association between *CHIT1* genotype and the intensity of hookworm infection as determined by faecal egg counts. The frequency of the mutant allele was 0.251, very similar to that found in non-endemic countries. The extent of geographical variation in allele frequencies across worldwide populations was not high (*F*_st_ = 0.11), and does not provide evidence for directional selection at this locus between different geographical areas. We conclude that the *CHIT1* genotype does not play a crucial role in protection against hookworm infection. This does not correlate with a previous study that linked the mutant *CHIT1* genotype to filariasis susceptibility. The possible reasons for this discrepancy are discussed.

## 1 Introduction

Chitotriosidase is a chitinolytic enzyme which was recently identified in man and is synthesised in large quantities by activated macrophages (Hollak et al., 1994). As humans lack endogenous chitin, a role for chitotriosidase is not fully understood. However the anti-fungal action of homologous plant chitinases has led to the suggestion that chitotriosidase may degrade chitin containing pathogens (Boot et al., 1998). The chitotriosidase gene (*CHIT1*) consists of 12 exons located on chromosome 1q31–32 (Boot et al., 1998). A 24 base pair duplication in exon 10 leads to the activation of a cryptic 3′ splice site that results in an abnormally spliced mRNA with an inframe 87 nucleotide deletion (Boot et al., 1998). The mutant protein lacks amino acids 344–372 that are required for the formation of the TIM-barrel catalytic core (Boot et al., 1998; Fusetti et al., 2002). Macrophages from chitotriosidase deficient individuals express only small amounts of mRNA and secrete virtually no chitotriosidase protein (Boot et al., 1998).

In Dutch and Ashkenazi Jewish populations 6% of individuals are homozygous for the mutant allele, whereas 35% and 34%, respectively, are heterozygous carriers (Boot et al., 1998) with a mutant allele frequency of 0.23. A similar allelic frequency is present in Portugal (0.22), Sicily (0.27) and Sardinia (0.21) (Malaguarnera et al., 2003). However in two West African countries, Benin and Burkina Faso, that are mesoendemic for *Plasmodium falciparum* malaria and endemic for gastrointestinal helminths, a total absence of the homozygous mutation and significant reduction in heterozygous individuals has been reported, with mutant allele frequency 0.00 and 0.02, respectively (Malaguarnera et al., 2003). This observation led to the hypothesis that chitotriosidase may be involved in resistance to protozoan or helminth infections common in tropical countries (Malaguarnera et al., 2003).

Parasitic nematodes are known to contain chitin, and are thus a potential target of human chitotriosidase. Chitin is found in the egg-shell of both free-living and parasitic nematodes, and in the microfilarial sheath surrounding the first-stage larvae of filarial nematodes (Fuhrman and Piessens, 1985). More recently, chitin has been demonstrated in the pharynx of both the free-living *Caenorhabditis elegans* (Zhang et al., 2005) and the gastrointestinal parasite *Oesophagostomum dentatum* (Neuhaus et al., 1997). There are two chitin synthase genes in *C. elegans*, *chs-1* and *chs-2*, encoding eggshell and pharynx chitin, respectively, and *chs-2* knockdown leads to lack of pharyngeal function and starvation (Zhang et al., 2005). This suggests that host chitotriosidase could potentially interfere with parasitic nematode feeding. Two studies to date have investigated associations between *CHIT1* genotype and filarial nematode infection. Choi et al. (2001) found significant association between susceptibility to lymphatic filariasis (*Wuchereria bancrofti*) and homozygosity for the mutant allele in an Indian population, but in a similar study in Papua New Guinea, no association between *CHIT1* genotype and lymphatic filariasis was observed (Hise et al., 2003).

There have been no previous studies investigating *CHIT1* genotype and gastrointestinal nematode infection. Human hookworm infection is an abundant chronic gastrointestinal nematode infection in sub-tropical and tropical countries, causing significant morbidity, principally due to iron-deficiency anaemia (Brooker et al., 2004). Human hookworm burden (as assessed by faecal egg count) is known to be under host genetic control (Williams-Blangero et al., 1997), but the genes responsible have not been identified. The aims of the current study were (1) to investigate associations between *CHIT1* genotype and human hookworm infection in an endemic population from Madang Province, Papua New Guinea and (2) to investigate evidence for directional selection at the *CHIT1* locus, by comparing worldwide allele frequency variation at the *CHIT1* locus to that observed at other loci across the genome.

## 2 Materials and methods

### 2.1 Geographical location and study population

The study population consisted of five villages in lowland Madang Province, Papua New Guinea, where *Necator americanus* is the only hookworm species (Pritchard et al., 1990). Villages were censused and pedigree information collected in 1998, and sample containers for faecal collection offered to all individuals aged 4 years and above. Faecal egg counts were performed using a modified McMaster salt flotation method and results were expressed as eggs per gram (epg) of faeces (Quinnell et al., 2004). Venous blood was then collected, and all individuals were offered treatment with albendazole or pyrantel pamoate. DNA was extracted from buffy coats using phenol/chloroform. In September 2001, a second faecal sample was taken from three of the five villages and reinfection hookworm burden determined; some previously untreated individuals provided samples in 2001. Hookworm epg has been shown to be a heritable phenotype in the study population (Breitling and Quinnell, unpublished results). The study was approved by the Medical Research Advisory Committee of Papua New Guinea and informed consent was received from all subjects or their parents.

### 2.2 Chitotriosidase genotyping by PCR

Thermal cycling reactions consisted of Reddy Mix PCR master mix (ABgene), 4.5 pmol forward and 4.5 pmol reverse chitotriosidase primers (forward: AGCTATCTGAAGCAGAAG; reverse: GGAGAAGCCGGAAAGTC) (Sigma) and 2 μl genomic DNA in a final volume of 15 μl. Samples were run in 96 well PCR plates (ABgene) on a Peltier PTC-200 Thermal Cycler (MJ Research). The thermal cycling protocol was: 94 °C for 5 min; 35 cycles of 94 °C for 30 s, 54 °C for 30 s and 72 °C for 30 s; and 72 °C for 5 min. Thermal cycling products with 10 base pair markers (Promega) were separated on 4% high resolution agarose (Sigma) gels in 1× TBE buffer and ethidium bromide. Gels were viewed using the Gene Genius Bio-imaging System and Gene Snap software (Syngene). The size of wild type product is 75 base pairs whereas the mutant product is 99 base pairs due to the mutant allele containing a 24 base pair duplication in exon 10 (Boot et al., 1998).

### 2.3 Statistical analysis

Variation in faecal egg counts by *CHIT1* genotype was analysed under both a codominant and recessive model. Faecal egg counts were highly overdispersed, so analysis was performed using a generalised linear model with a negative binomial error structure in Stata 9.1. This method has been shown to be more accurate than analysis of log-transformed parasite burden data (Wilson et al., 1996). Significant covariates included in the analysis were age, village, faecal consistency, anthelmintic treatment and the age × treatment and village × treatment interaction terms. Egg counts from both years were analysed together; since some individuals were sampled both pre- and post-treatment, standard errors were adjusted for the non-independence of samples from the same individual using the ‘cluster’ option. To control for non-independence of individuals due to genetic relatedness, further analysis was performed with the total test of association in a variance components framework using the programme QTDT (Abecasis et al., 2000). Finally, to control for potential population stratification, transmission disequilibrium testing was carried out using the orthogonal association model in QTDT. The phenotypic variable used for these analyses was the residuals from a negative binomial regression of egg counts against significant covariates; where two samples were taken from the same individual, the mean residual for that individual was calculated.

To quantify geographic variation in allele frequencies, *F*_st_ values were calculated according to Cockerham and Weir (1984) using the programme FSTAT 2.9.3 (Goudet, 1995). Allele frequencies were compared across five broad geographical areas, using the weighted mean allele frequency for each geographical area (Europe/Mediterranean, Africa, South Asia, East Asia and Papua New Guinea). To compare observed values of *F*_st_ to those expected for neutral markers, *F*_st_ was also calculated across comparable populations selected from the Human Genome Diversity Panel using published data for 210 genome-wide short insertion/deletion polymorphisms genotyped by the Mammalian Genotyping Service (data available from http://research.marshfieldclinic.org/genetics/) (Soranzo et al., 2005). The populations selected were European (French/Italian), African (Yoruba), South Asia (Sindhi), East Asia (Chinese Han) and Papuan.

## 3 Results

In total 693 individuals from Papua New Guinea were genotyped for the chitotriosidase mutation. The allelic frequency of the 24 bp duplication in 279 unrelated individuals, determined by PCR genotyping, was 0.251. The percentage of individuals who were homozygous mutant was 6% (16/279), heterozygous 39% (108/279) and homozygous wild type 56% (155/279). Genotype frequencies did not differ from those expected under Hardy–Weinberg equilibrium (*χ*^2^ = 0.25, d.f. = 1, *P* = 0.62).

Both egg counts and *CHIT1* genotypes were available for a total of 602 individuals (574 sampled pre-treatment and 210 sampled post-treatment). The prevalence and intensity of infection (mean egg count) in these individuals were 81% (2574 epg) pre-treatment, and 92% (1727 epg) post-treatment. Egg counts were very similar across *CHIT1* genotypes both pre-treatment and after reinfection (Table 2). Combined analysis of both years’ data revealed no significant relationship between egg count and genotype under either a codominant (*χ*^2^ = 0.03, d.f. = 1, *p* = 0.87) or recessive model (*χ*^2^ = 0.02, d.f. = 1, *p* = 0.90). Further analysis using the total test of association in QTDT, controlling for non-independence of individuals due to genetic relatedness, again showed no significant association between *CHIT1* genotype and egg count (*χ*^2^ = 0.39, d.f. = 1, *p* = 0.53). Similarly, transmission disequilibrium testing, which controls for population stratification, was not significant (*χ*^2^ = 0.58, d.f. = 1, *p* = 0.45).

Published allele frequencies for *CHIT1* in different geographical areas are shown in Table 1. Using these data and data from the current study, *F*_st_ values were calculated to measure variation in allele frequency across five broad geographical areas (Europe, Africa, South Asia, East Asia, and Papua New Guinea). The *F*_st_ across all populations for *CHIT1* was 0.11. This was comparable to the median *F*_st_ of 0.12 for 210 indels typed in populations from similar geographical areas. Pairwise comparisons showed higher *F*_st_ values between Africa and East Asia (*F*_st_ = 0.69) and Africa and South Asia (*F*_st_ = 0.54), than those between other areas (*F*_st_ < 0.40). In each case the observed *F*_st_ was greater than that for all but 7 of 210 indels, giving an empirical *P* = 0.038 (8/210).

## 4 Discussion

The results from the present study in Papua New Guinea strongly suggest that chitotriosidase does not have a critical role in protection against *N. americanus*. The relative number of people who are wild type homozygous for chitotriosidase, heterozygous and homozygous mutant is similar to that seen in countries where this infection is not endemic, suggesting no advantage for the wild type allele in endemic areas. Moreover, there was no evidence for an association between *CHIT1* genotype and hookworm burden in our study population.

The mutant chitotriosidase genotype has been associated with an increased susceptibility to *W. bancrofti* infection in a study of 216 individuals from South India (Choi et al., 2001). Previously it has been demonstrated that the microfilariae, oocytes and zygotes of the filarial nematode *Brugia malayi* contain chitin (Fuhrman and Piessens, 1985; Schraermeyer et al., 1987). In the rodent filarial nematode *Acanthocheilonema vitae,* there are distinctive chito-oligomeric *N-*glycans that contain projections of up to six GlcNAc residues that may be involved in parasite-host interactions (Haslam et al., 1999). Additionally chitin synthase is required for sheath development in microfilarial progeny. Chitin has also been identified in the cuticle of mature *W. bancrofti* (Araujo et al., 1993). The presence of chitin in the sheath and cuticle of filarial nematodes and the requirement for chitin synthase in reproduction show that chitin has an important role in the lifecycle of these species. If host-derived chitotriosidase can access this chitin, thus interfering with the parasite's growth and development, then it may explain why susceptibility to *W. bancrofti* is increased in individuals with the mutant genotype. However, a second study by Hise and coauthors (Hise et al., 2003) in 906 individuals in a region endemic for bancroftian filariasis in Papua New Guinea did not find any significant correlation with infection status or disease phenotype. Thus the issue is in further need of clarification.

In contrast to filarial worms, gastrointestinal nematodes such as hookworm do not have a microfilarial stage, and the chitin-containing eggs are not accessible to host chitotriosidase. However, chitin has recently been demonstrated in the pharynx of the free-living nematode *C. elegans* (Zhang et al., 2005). It is not known whether chitin occurs in the pharynx of *N. americanus*, but it has been demonstrated in the related strongyloid parasite *O. dentatum* (Neuhaus et al., 1997). Pharyngeal chitin provides a potential target for host chitotriosidase, both in tissue-dwelling larval stages, which may be accessible for macrophage or neutrophil-derived chitotriosidase, and blood-feeding adults. Chitotriosidase levels can be elevated in serum, e.g. in acute falciparum malaria (Barone et al., 2003), and as adult hookworms are thought to ingest 0.03–0.2 ml of blood daily, they may be exposed to high levels of chitotriosidase. However, the lack of an association between *CHIT1* genotype and hookworm burden seen in the present study suggests that chitotriosidase is not an important effector mechanism against hookworm infection. Further studies would be useful to examine the bioavailability of nematode chitin, its accessibility for exogenous chitinases, or their effect on parasite viability.

The mutant allele frequency observed in the current study was higher than that in another Papuan population from East Sepik Province (Hise et al., 2003). Such variation within Papua New Guinea is not surprising, and differences between these populations have been observed at other loci (Main et al., 2001). Analysis of worldwide variation in allele frequencies showed that the degree of population differentiation at the *CHIT1* locus (*F*_st_ = 0.11) was very similar to the median value for 210 indels across the genome. Though the populations sampled were not identical, this suggests that there has not been directional selection at this locus across different geographical areas, and that the worldwide variation in allele frequencies is consistent with genetic drift (Akey et al., 2002). There was some evidence for higher than average population differentiation at the *CHIT1* locus between African and Asian populations, which may suggest some local adaptation, though this would not be significant after correction for multiple testing.

Malaguarnera et al. (2003) suggest that an intact *CHIT1* genotype may be advantageous in protection against malaria, based on the very low frequency of the mutant genotype in Burkina Faso and Benin, where malaria is *meso*-endemic. However, our results are not consistent with the hypothesis that chitotriosidase is involved in protection against malaria, or any tropical disease. There is no evidence for directional selection worldwide, and the greatest difference in allele frequencies is observed between Africa and other tropical countries, also endemic for malaria (Choi et al., 2001; Chien et al., 2005). Although Barone et al. (2003) found that chitotriosidase levels are elevated in the serum in acute *P. falciparum* malaria, there is no evidence that elevated serum chitotriosidase levels have an impact on the severity and outcome of malaria, and no study has compared genotypes with disease status. Indeed, increased serum chitotriosidase may actually increase malaria transmission, by inhibiting the formation of the peritrophic membrane in the anopheline gut (Di Luca et al., 2007). As the study area in Papua New Guinea is endemic for malaria, we have performed an association analysis which showed no significant difference between chitotriosidase genotype and the presence of *P. falciparum* and *P. vivax* malaria infection (data not shown). The caveat of this analysis is that it was based on a single time point assessment of parasitaemia, rather than repeated measurements. As parasitaemia is very variable, we cannot make a reliable statement regarding the effect of the *CHIT1* genotype on malarial infection, and further studies are needed.

Taken together, our data suggest that the chitotriosidase genotype does not play a major role in protection against hookworm infection, but this may be different in the case of lymphatic or cutaneous filariasis. However, there is no evidence for selection at this locus mediated by malaria or another tropical disease.

## Figures and Tables

**Table 1:** Genotype and allele frequencies of the *CHIT1* mutation in different populations

Population	Subjects	(%) wt/wt	(%) wt/mut	(%) mut/mut	Wt allele frequency	Mut allele frequency	Reference
Sicily	100	51	43	6	0.73	0.27	Malaguarnera et al. (2003)
Sardinia	107	64	32	4	0.79	0.21	Malaguarnera et al. (2003)
Portugal	295	60	37	3	0.78	0.22	Rodrigues et al. (2004)
Holland	171	59	35	6	0.77	0.23	Boot et al. (1998)
Ashkenazi Jews	68	60	34	6	0.77	0.23	Boot et al. (1998)
South India	67	31	58	10	0.60	0.40	Choi et al. (2001)
Papua New Guinea	906	77	22	1	0.88	0.12	Hise et al. (2003)
Chinese Han(Taiwan)	82	15	55	30	0.42	0.58	Chien et al. (2005)
Benin	100	100	0	0	1.00	0.00	Malaguarnera et al. (2003)
Burkina Faso	100	98	2	0	0.98	0.02	Malaguarnera et al. (2003)

wt: wild-type (intact) allele; mut: mutant allele (24 bp duplication).

**Table 2:** Hookworm burdens according to *CHIT1* genotype in the Papua New Guinea study population

Genotype	*CHIT1*			
	Pre-treatment		Post-treatment	
	*n*	Hookworm epg	*n*	Hookworm epg
wt/wt	320	2495 (2109–2952)	102	1636 (1259–2127)
wt/mut	222	2675 (2131–3357)	98	1815 (1436–2295)
mut/mut	32	2374 (1377–4090)	10	1648 (882–3079)

Burdens were assessed as mean eggs per gram faeces (epg) (95% CL) before anthelmintic treatment and after reinfection. Confidence intervals were calculated assuming a negative binomial error distribution.
